# The effects of health worker motivation and job satisfaction on turnover intention in Ghana: a cross-sectional study

**DOI:** 10.1186/1478-4491-12-43

**Published:** 2014-08-09

**Authors:** Marc Bonenberger, Moses Aikins, Patricia Akweongo, Kaspar Wyss

**Affiliations:** 1 Swiss Centre for International Health, Swiss Tropical and Public Health Institute, Socinstrasse 57, Basel 4002, Switzerland; 2 University of Basel, Basel, Switzerland; 3 School of Public Health, University of Ghana, Legon, Ghana

**Keywords:** Motivation, Job satisfaction, Turnover intention, Retention, Health worker, Human resource management, Rural and remote areas, Ghana

## Abstract

**Background:**

Motivation and job satisfaction have been identified as key factors for health worker retention and turnover in low- and middle-income countries. District health managers in decentralized health systems usually have a broadened ‘decision space’ that enables them to positively influence health worker motivation and job satisfaction, which in turn impacts on retention and performance at district-level. The study explored the effects of motivation and job satisfaction on turnover intention and how motivation and satisfaction can be improved by district health managers in order to increase retention of health workers.

**Methods:**

We conducted a cross-sectional survey in three districts of the Eastern Region in Ghana and interviewed 256 health workers from several staff categories (doctors, nursing professionals, allied health workers and pharmacists) on their intentions to leave their current health facilities as well as their perceptions on various aspects of motivation and job satisfaction. The effects of motivation and job satisfaction on turnover intention were explored through logistic regression analysis.

**Results:**

Overall, 69% of the respondents reported to have turnover intentions. Motivation (OR = 0.74, 95% CI: 0.60 to 0.92) and job satisfaction (OR = 0.74, 95% CI: 0.57 to 0.96) were significantly associated with turnover intention and higher levels of both reduced the risk of health workers having this intention. The dimensions of motivation and job satisfaction significantly associated with turnover intention included career development (OR = 0.56, 95% CI: 0.36 to 0.86), workload (OR = 0.58, 95% CI: 0.34 to 0.99), management (OR = 0.51. 95% CI: 0.30 to 0.84), organizational commitment (OR = 0.36, 95% CI: 0.19 to 0.66), and burnout (OR = 0.59, 95% CI: 0.39 to 0.91).

**Conclusions:**

Our findings indicate that effective human resource management practices at district level influence health worker motivation and job satisfaction, thereby reducing the likelihood for turnover. Therefore, it is worth strengthening human resource management skills at district level and supporting district health managers to implement retention strategies.

## Background

Improving the retention of the health workforce in rural and remote areas is of concern for all countries worldwide [1]. Improved retention of health workers contributes to the provision of quality health care because it builds up competencies, optimizes team relations, and strengthens the relationship of health workers with local communities [2]. In contrast, poor retention or high staff turnover negatively affects health care by increasing workload, undermining team morale, creating disruptions and inefficiencies in work processes, and causing a loss of institutional knowledge [3].

Several factors influence the decision of health workers to stay in or leave their posts. Among these are low pay, poor career structures, lack of opportunities for postgraduate training, and inadequate working and living conditions [4-7]. The challenge of retaining health workers is greatest in rural and remote areas, because health practitioners in these areas often face higher workloads, unsustainable work environments, and poor infrastructure, causing them to leave the workplace in search of more satisfactory working and living conditions in urban areas or abroad [8].

This study was carried out within the framework of the health human resource management (HRM) intervention ‘Supporting decentralized management to improve health workforce performance in Ghana, Uganda and Tanzania (PERFORM)’. The intervention aims at identifying ways of strengthening district management in order to address health workforce inadequacies by improving health workforce performance in sub-Saharan Africa. By taking into account national and local human resource (HR) and health system (HS) policies and practices already in place, ‘bundles’ of HR/HS strategies - such as task shifting, training, supervision and monitoring - are developed with respect to specific health workforce problems in the study districts. These strategies must be feasible within the context and affordable within the districts’ budgets to strengthen priority areas of health workforce performance [9].

Typically, an important share of HRM activities takes place at district level. In countries with implemented health sector decentralization policies, district health managers have, as Bossert [10] calls it, a broadened ‘decision space’, which refers to effective decision-making or range of choice within the various functions of finance, service organization, HR, targeting and governance. Although key functions of HRM - such as recruitment, remuneration patterns, and promotion - often remain highly centralized [11], district health managers in decentralized contexts have the potential to improve retention and performance outcomes in their districts. Their powers include exact posting and performance management such as in-service training, supportive supervision, and staff appraisal [12-14]. Generally, it is assumed that these aspects result in lower turnover respectively improved retention at district level.

Work motivation and job satisfaction have been identified as key factors of health worker retention and turnover in low- and middle-income countries (LMICs) [15-21]. Motivation has been described as a set of psychological and transactional processes: psychological, because it gives behaviour purpose and direction; transactional, because it is the result of the interactions between individuals and their work environment [22]. The conceptual framework of health worker motivation proposed by Kanfer [23] and Franco *et al*. [24] describes these motivational processes as being affected by determinants of motivation (for example, incentives, values, expectations) and mediated into motivational outcomes such as performance and job satisfaction.

As motivation *per se* cannot be observed directly [25], previous research has concentrated on the determinants and outcomes of motivation [13,15,26]. Willis-Shattuck *et al*. [15] conducted a systematic review on motivation and retention in LMICs and identified monetary and non-monetary incentives as core factors affecting motivation and retention in these countries. This is in line with the World Health Organization (WHO), which recommended a mix or ‘bundle’ of interventions in the areas of education, regulation, financial incentives, and professional and personal support in order to improve retention of health workers in rural and remote areas [1]. Many of these interventions are targeting at improving health worker motivation, job satisfaction and performance.

Job satisfaction is a frequently studied motivational outcome in health systems research. Due to its relation to performance and turnover, it is of concern to researchers and health service managers alike [27-29]. As Lu *et al*. [26] have pointed out, job satisfaction depends both on the nature of the job and on the expectations health workers have of what their job should provide, and is thus the affective orientation that employees have towards their work. Job satisfaction can be measured globally or in a multi-faceted way, with the former approach being used when the interest is on overall attitude toward the job, and the latter when specific aspects of job dissatisfaction are evaluated [28]. Important facets of job satisfaction having been identified in previous research include salary and benefits, career development, in-service training, work relationships, management, work environment, recognition and supervision [19,30-32].

So to explore health worker motivation and job satisfaction and their effects on turnover intention, we conducted a cross-sectional survey in three districts of the Eastern Region in Ghana. A better knowledge of factors that impact on workforce performance was needed in order to inform district health managers who are essential at the operational level of health systems and important drivers for performance outcomes in their districts.

## Methods

### Study setting

The study was carried out in the Eastern Region of Ghana in the Akwapim North, Upper Manya Krobo and Kwahu West districts. Akwapim North is a mostly rural district also containing some towns and is located in the proximity of Ghana’s capital, Accra, as well as the region’s capital Koforidua. Subsistence and commercial farming is the predominant occupation, although manufacturing, extractive industries and small-scale industries also exist [33]. In Kwahu West, around 50% of the population is concentrated in the district capital Nkawkaw, a well-known commercial town. The rest of the settlements are small communities located mainly along the Accra-Kumasi Highway that crosses the district. Although about 50% of the population are subsistence farmers, trade and commerce are also important occupations [34]. Upper Manya Krobo is predominantly rural and is regarded as one of the highly underdeveloped districts in the Eastern Region with poor infrastructure. The district comprises Asesewa, a small town and the capital of the district, and 198 rural communities. Around 80% of the population consists of subsistence farmers and fishermen, the rest mainly engage in commerce and small-scale industries [35].

A total of 939 public and private non-profit health workers were working in the study districts during data collection in 2012, including administrative and support workers such as accountants and labourers. The majority of these health workers were working in two public hospitals and one private faith-based non-profit hospital. The rest were distributed between the 22 health centres, and 37 Community-based Health Planning and Services (CHPS) facilities.

CHPS is a primary health care programme in Ghana, which was pilot-tested in the early 1990s and then rolled-out as a national policy from the late 1990s onwards in order to increase health service accessibility of people living in rural and remote areas [36,37]. CHPS facilities are staffed by community health nurses (CHN) who provide mobile doorstep health services to community residents.

### Data collection instrument

We used a structured questionnaire to collect quantitative data on motivation, job satisfaction and turnover intention. In order to measure turnover intention we asked health workers, whether they had intentions to leave their current health facility, for which only dichotomous responses (yes/no) were possible. Job satisfaction was measured with an instrument, which - on the basis of the Measure of Job Satisfaction (MJS) [38] and the Job Descriptive Index (JDI) [39] - was originally developed by Fournier *et al*. [40] for application in Mali. The tool was then developed further and validated for research on job satisfaction of various types of health care workers in francophone Western Africa [19,41]. For the present study we used the version published by Rouleau *et al*. [19]. As the instrument was originally written in French, we translated the questions into English. The instrument was composed of 39 5-point Likert-scale items, ranging from 1, very unsatisfied, to 5, very satisfied, and were grouped into 9 facets of job satisfaction, namely ‘remuneration’, ‘work environment’, ‘workload’, ‘tasks’, ‘working relations’, ‘in-service training’, ‘management’, ‘morale’, and ‘job security’.

For the measurement of motivation, we used the instrument from Mbindyo *et al*. [42], who adapted it from Bennett *et al*. [25] and Kanfer [23] to measure motivation in district hospitals in Kenya. The instrument was recently validated by Mutale *et al*. [43] for the use at community level in Zambia. The instrument was composed of 23 5-point Likert-scale items, ranging from 1, fully disagree, to 5, fully agree, and where grouped into 7 motivational outcome constructs, namely ‘general motivation’, ‘burnout’, ‘job satisfaction’, ‘intrinsic job satisfaction’, ‘organizational commitment’, ‘conscientiousness’, and ‘timeliness’. For the ‘job satisfaction’ construct, we used the overall job satisfaction score instead of the original items of the motivation instrument as described by Mbindyo *et al*. [42]. This was done in order to decorticate the relationship between motivation and job satisfaction in our motivation construct, where job satisfaction is an outcome.

We pretested the questionnaire in the Eastern Regional Hospital in Koforidua, during which we focused particularly on the comprehensibility and relevance of the questions for all types of health workers included in the study. The questionnaire was reviewed after the pretest, mainly to localize some of the demographic and work-related questions to the Ghanaian context. In order to ensure consistency across interviewers the pretest was also used to train field assistants on interview techniques.

### Sampling strategy and data collection

The sampling was based on a staff inventory such as established by District Health Management Teams (DHMTs). We did not include the administrative and support workforce and based the sampling on a list containing 626 clinical, nursing, midwifery, and pharmacy staff, and the allied health staff. Based on Cochran’s sample size formula for categorical data we estimated the required sample size to be around 300.

In order to receive a representative sample of the three districts, we applied a systematic sampling strategy for which we reordered the list alphabetically by health facility and then selected every second health worker from this list. When a health worker was not present at a health facility when the study team arrived or refused to participate in the study, we selected the next person from the list. When this person was also not available for one or the other reason we did not choose another participant.

Data collection was carried out between September and November 2012 by the lead author and two field assistants. In total, we interviewed 256 health workers in 59 of the 62 (95%) health facilities in the three study districts. The majority of the interviewed health workers were working in the 3 district hospitals (n = 158), the rest worked in health centres and CHPS facilities (n = 98) at subdistrict level. Most of the visited facilities were public health facilities of the Ghana Health Service (GHS). Only the district hospital of Kwahu West belonged to the Christian Health Association of Ghana (CHAG), a faith-based private non-profit organization.

### Statistical analysis

We entered the data using Epi Info 7 and used STATA 13 (STATA Corp., College Station, TX, USA) for the statistical analysis. Because we were only interested in voluntary turnover, we excluded from the analysis health workers who expressed turnover intention due to retirement (n = 28). Missing data and ‘do not know’-responses were imputed with the mean score of the respective items. As some of the statements of the motivation instrument were negatively worded, we reversed responses to negative items so that higher scores indicated disagreement to these statements.

We conducted two separate factor analyses in order to confirm the latent factors reported by Rouleau *et al*. [19] and Mbindyo *et al*. [42]. The nine latent factors of the job satisfaction instrument as well as the seven factors of the motivation instrument were confirmed. However, items loading under two factors of the job satisfaction instrument could rather be described as ‘career development’ and ‘supervision’, for which reason we dropped ‘job security’ and ‘working relations’ as used in the original instrument and retained the newly identified factors (see Additional files 1 and 2 for the results of the factor analyses).

In the univariate analysis we tested for statistically significant associations using Chi-square tests for binary and categorical variables. The Wilcoxon rank-sum test and the Kruskal-Wallis test were used to examine differences of numeric variables between two or more groups, as appropriate. Spearman’s rank correlation was used to assess the relationship between turnover intention and the overall motivation and job satisfaction scales as well as their respective subscales. We also conducted several univariable logistic regression analyses to assess associations of turnover intention with motivation, job satisfaction, and demographic and work-related characteristics.

For the multivariate analysis we fitted several multiple logistic regression models by applying a combination of forward stepwise logistic regression and purposeful selection of variables. We included all variables in the forward stepwise regressions, which we found to be significantly associated with turnover intention in the univariate analysis. The significance level for entering a variable into the models was set to 0.15, and to 0.2 for removal from the models. We then fitted logistic regression models by including all variables that had been found through stepwise selection and assessed the significance of these variables through Wald-*P* statistics. All variables with *P* ≥ 0.05 were eliminated from the models. We controlled for confounding by re-entering all excluded variables, which had marked effects on the coefficients ( $\Delta \hat{\beta }$ > 20%) of the variables remaining in the models. In all multiple logistic regression models, profession, type of facility and district were retained as fixed effects.

### Ethical considerations

As PERFORM is coordinated by the Liverpool School of Tropical Medicine (LSTM), ethical clearance for the whole study was obtained from the Research Ethics Committee of LSTM (ID Number: 12.09). For the present study we obtained additional ethical clearance from the Ghana Health Service Ethical Review Committee (ID Number: GHS-ERC: 13/05/12). We sought written informed consent before interviewing any of the respondents. We anonymized all personal data by assigning ID numbers to the study participants. Only these numbers were used on the questionnaires, for data entry, and in the subsequent analysis instead of names.

## Results

### Characteristics of the health workforce

Table 1 presents the characteristics of the health workforce, with comparisons made between districts. The majority of the health workers consisted of females (82.4%) and persons aged under 40 years (64.9%). There was a noticeable shortage of middle-aged health workers in Upper Manya Krobo, as most respondents were below 30 years of age (67.9%), indicating problems with attraction and retention of experienced health professionals. Work experience was rather low as median time spent in the profession by health workers across all districts was four years, ranging between two years (Upper Manya Krobo) and six years (Kwahu West). The majority of all health workers worked 3 years or less in their current health facility (53.2%), with the majority of respondents from Upper Manya Krobo working only 1 year or less in their present facility (50.9%). About 72% of the health workforce working in the 3 study districts had a certificate, 18% had a diploma and 10% had earned a higher degree (bachelor’s, master’s or doctoral degree). About 62% of the health workforce worked in one of the three district hospitals and 38.3% in health centres or CHPS facilities. With 82.9%, the nursing and midwifery professions were the most available cadres in the districts’ health systems and doctors were the least available with 4.3% of the workforce.

**Table 1:** Characteristics of the health workforce

		**District, n (%)**			
**Demographic and work-related characteristics**	**All respondents (n = 256), n (%)**	**Akwapim North (n = 91, 36%)**	**Upper Manya Krobo (n = 53, 21%)**	**Kwahu West (n = 112, 44%)**	***P***^**b**^
Female gender	211 (82.4)	74 (81.3)	43 (81.1)	94 (83.9)	0.855
Age (years)					
< 30	121 (47.3)	37 (40.7)	36 (67.9)	48 (42.9)	**0.038**^**a**^
30 to 39	45 (17.6)	17 (18.7)	6 (11.3)	22 (19.6)	
40 to 49	23 (9.0)	9 (9.9)	1 (1.9)	13 (11.61)	
≥ 50	67 (26.2)	28 (30.8)	10 (18.87)	29 (25.9)	
Marital status					
Single	108 (42.4)	39 (42.9)	36 (67.9)	33 (29.7)	**< 0.001**^**a**^
Married	128 (50.2)	46 (50.6)	15 (28.3)	67 (60.4)	
Divorced/widowed	19 (7.5)	6 (6.6)	2 (3.8)	11 (9.9)	
Qualification					
Certificate	183 (71.5)	68 (74.7)	43 (81.1)	72 (64.3)	**0.005**^**a**^
Diploma	47 (18.4)	9 (9.9)	9 (17.0)	29 (25.9)	
Higher	23 (10.2)	14 (15.4)	1 (1.9)	11 (9.8)	
Years in profession, median	4	4	2	6	**0.001**^**ac**^
Years working in current health facility					
≤ 1	79 (30.9)	33 (36.3)	27 (50.9)	19 (17.0)	**< 0.001**^**a**^
> 1 and ≤ 3	57 (22.3)	20 (22.0)	15 (28.3)	22 (19.6)	
> 3 and ≤ 5	35 (13.7)	11 (12.1)	5 (9.4)	19 (17.0)	
> 5	85 (33.2)	27 (29.7)	6 (11.3)	52 (46.4)	
Type of health facility					
Hospital	158 (61.7)	50 (55.0)	24 (45.3)	84 (75.0)	**< 0.001**^**a**^
Health centre/CHPS	98 (38.3)	41 (45.0)	29 (54.7)	28 (25.0)	
Profession					
Doctors	11 (4.3)	4 (4.4)	1 (1.9)	6 (5.3)	**0.004**^**a**^
Nursing professions	212 (82.9)	74 (81.4)	49 (92.5)	89 (79.4)	
Registered nurses	48 (18.8)	17 (18.7)	9 (17.0)	22 (19.6)	
Midwives	34 (13.3)	11 (12.1)	7 (13.2)	16 (14.3)	
Auxiliary nurses	34 (13.3)	16 (17.6)	10 (18.9)	8 (7.1)	
CHNs	63 (24.6)	21 (23.1)	22 (41.5)	20 (17.9)	
Health care/ward assistants	33 (12.9)	9 (9.9)	1 (1.9)	23 (20.5)	
AHW/Pharmacists	33 (12.9)	13 (14.2)	3 (5.7)	17 (15.2)	

^a^statistical significance *P* < 0.05; ^b^Chi-square test; ^c^Kruskal-Wallis test.

CHPS: Community-based Health Planning and Services; CHN: community health nurse; AHW: allied health worker.

### Motivation and job satisfaction mean scores and turnover intention

Table 2 shows the overall motivation and job satisfaction mean scores as well as the means of their respective subscores, with comparisons made between persons with and without turnover intention. Health workers achieved an overall motivation mean score of 3.65 (out of 5). With regard to the motivation subscores, the lowest mean scores were reached in job satisfaction (3.15) and burnout (3.29) and the highest mean scores in timeliness and attendance (4.15), and conscientiousness (4.35). Concerning job satisfaction, health workers achieved an overall job satisfaction mean score of 3.15 (out of 5). The least job satisfaction mean subscores were remuneration (2.12) and career development (2.58). The highest mean subscores were reached in the areas of supervision (3.81) and morale (3.85).

**Table 2:** Motivation and job satisfaction mean scores and their relation to turnover intention

		**Turnover intention, mean ± SD**		
**Scales and subscales**	**Total (n = 228) mean ± SD**	**Yes (n = 157, 69%)**	**No (n = 71, 31%)**	***P***^**c**^
Overall motivation score	3.65 ± 0.38	3.58 ± 0.38	3.81 ± 0.31	**< 0.001**^**a**^
Job satisfaction	3.15 ± 0.46	3.08 ± 0.49	3.30 ± 0.37	**0.001**^**a**^
Burnout	3.29 ± 0.99	3.20 ± 0.99	3.49 ± 0.95	**0.02**^**a**^
General motivation	3.30 ± 0.86	3.21 ± 0.88	3.52 ± 0.77	**0.008**^**a**^
Organizational commitment	3.31 ± 0.75	3.14 ± 0.76	3.68 ± 0.57	**< 0.001**^**a**^
Intrinsic job satisfaction	4.02 ± 0.51	3.97 ± 0.57	4.13 ± 0.35	**0.036**^**a**^
Timeliness and attendance	4.15 ± 0.53	4.13 ± 0.53	4.20 ± 0.54	0.263
Conscientiousness	4.35 ± 0.42	4.35 ± 0.43	4.35 ± 0.41	0.929
Overall job satisfaction score^b^	3.15 ± 0.46	3.08 ± 0.49	3.30 ± 0.37	**0.001**^**a**^
Remuneration	2.12 ± 0.72	2.07 ± 0.69	2.25 ± 0.77	0.16
Career development	2.58 ± 0.94	2.47 ± 0.93	2.82 ± 0.94	**0.008**^**a**^
Management	2.76 ± 0.83	2.65 ± 0.85	2.98 ± 0.75	**0.008**^**a**^
Work environment	2.87 ± 0.76	2.79 ± 0.75	3.06 ± 0.76	**0.012**^**a**^
Workload	3.12 ± 0.79	3.07 ± 0.83	3.21 ± 0.69	0.304
In-service training	3.53 ± 0.90	3.52 ± 0.92	3.56 ± 0.86	0.843
Tasks	3.67 ± 0.71	3.61 ± 0.74	3.81 ± 0.61	**0.028**^**a**^
Supervision	3.81 ± 0.71	3.75 ± 0.74	3.94 ± 0.64	0.085
Morale	3.85 ± 0.63	3.75 ± 0.67	4.06 ± 0.46	**0.001**^**a**^

^a^statistical significance *P* < 0.05;

^b^all subscales refer to satisfaction with the dimensions under review;

^c^Wilcoxon rank-sum test.

Maximum score is 5. A higher score indicates higher levels of motivation and job satisfaction.

Overall, 69% (95% CI, 63 to 75) of the respondents in the 3 study districts reported to have intentions to leave their current health facility. When comparing the mean job satisfaction and motivation scores and subscores according to turnover intention, significant differences (*P* < 0.05) indicated that job satisfaction and motivation was lower for health workers having turnover intentions than for those not having turnover intentions.

### Determinants of turnover intention

Table 3 shows the crude odds ratios for sociodemographic and work-related factors associated with intention to leave the current health facility. The district was significantly associated with turnover intention, with the odds of having this intention being 5.11 times (95% CI: 2.05 to 12.73) greater for health workers from Upper Manya Krobo than for health workers from Akwapim North (baseline). For health workers from Kwahu West the odds were 1.87 times (95% CI: 1.00 to 3.47) greater than for those working in Akwapim North. Living away from one’s family (OR = 4.73, 95% CI: 2.45 to 9.15) and working in a health centre or CHPS facility (OR = 2.02, 95% CI: 1.10 to 3.67) also increased the odds, while being divorced or widowed (OR = 0.15, 95% CI: 0.04 to 0.52) decreased the odds of turnover intention. Being between 40 and 49 years of age (OR = 0.15, 95% CI: 0.06 to 0.39) and 50 years of age or older (OR = 0.16, 95% CI: 0.07 to 0.36) considerably decreased the odds, compared to those being younger than 30 years of age. Also working longer than 5 years (OR = 0.12, 95% CI: 0.06 to 0.26) in the current health facility considerably decreased the odds of intention to leave, compared to those working only 1 year or less in the present health facility. With regard to profession only the results for the health and ward assistants were statistically significant at a 5% level. Members of this cadre were considerably less likely of having turnover intentions (OR = 0.23, 95% CI: 0.08 to 0.62) than the registered nurse workforce (baseline).

**Table 3:** Crude odds ratios (ORs) for the effect of sociodemographic and work-related factors on turnover intention

**Sociodemographic and work-related factors**	**OR**	**95% CI**	***P***^**b**^
Female	1.08	0.53 to 2.21	0.824
Age group			
< 30 years	1	−	−
30 to 39 years	0.73	0.32 to 1.64	0.442
40 to 49 years	0.15	0.06 to 0.39	**< 0.001**^**a**^
≥ 50	0.16	0.07 to 0.36	**< 0.001**^**a**^
Marital status			
Single	1	−	−
Married	0.68	0.38 to 1.24	0.213
Divorced/widowed	0.15	0.04 to 0.52	**0.003**^**a**^
Living away from family	4.73	2.45 to 9.15	**< 0.001**^**a**^
Years working in current health facility			
≤ 1	1	−	−
> 1 and ≤ 3	0.86	0.36 to 2.07	0.739
> 3 and ≤ 5	0.80	0.29 to 2.21	0.667
> 5	0.12	0.06 to 0.26	**< 0.001**^**a**^
Profession			
Registered nurses	1	−	−
Doctors	1.23	0.28 to 5.44	0.777
Midwives	0.93	0.32 to 2.62	0.888
Auxiliary nurses	1.52	0.52 to 4.45	0.440
CHNs	2.19	0.87 to 5.53	0.096
Health/ward assistants	0.23	0.08 to 0.62	**0.003**^**a**^
AHW/Pharmacists	1.31	0.42 to 4.11	0.637
Type of facility			
Hospital	1	−	−
Health centre/clinic	2.02	1.10 to 3.67	**0.022**^**a**^
District			
Akwapim North	1	−	−
Upper Manya Krobo	5.11	2.05 to 12.73	**< 0.001**^**a**^
Kwahu West	1.87	1.00 to 3.47	**0.049**^**a**^

^a^statistical significance *P* < 0.05; ^b^Wald-test.

AHW: allied health worker; CHN: community health nurse; CHPS: Community-based Health Planning and Services.

### The effect of motivation and job satisfaction on turnover intention

Table 4 shows the results of the multivariable logistic regression including the overall motivation and job satisfaction scales as well as demographic and work-related factors. Motivation (OR = 0.74, 95% CI: 0.60 to 0.92) and job satisfaction (OR = 0.74, 95% CI: 0.57 to 0.96) were both significantly associated with turnover intention, indicating that health workers with higher levels of motivation and job satisfaction were less likely to having intentions to leave their current health facilities. As we found in the univariable model, also working more than 5 years (OR = 0.08, 95% CI: 0.02 to 0.32) in the present facility considerably decreased the odds of having turnover intention. The odds of turnover intention for health workers in Upper Manya Krobo (OR = 5.92, 95% CI: 2.10 to 16.67) were almost 6 times greater than the odds for health workers in Akwapim North, which resembled the result of the univariable model. The odds of turnover intention for health workers in Kwahu West (OR = 4.36, 95% CI: 2.00 to 12.31) increased in the multivariable model and were around 4 times greater than for those working in Akwapim North. Age, profession and type of health facility were not significantly associated with turnover intention.

**Table 4:** Adjusted odds ratios (AORs) for the effect of motivation and job satisfaction on turnover intention

**Risk determinants of turnover intention**	**AOR**	**95% CI**	***P***^**b**^
Motivation score	0.74	0.60 to 0.92	**0.006**^**a**^
Job satisfaction score	0.74	0.57 to 0.96	**0.025**^**a**^
Age^c^	0.91	0.71 to 1.16	0.444
Years working in current health facility			
≤ 1	1	−	−
> 1 and ≤ 3	0.45	0.14 to 1.44	0.179
> 3 and ≤ 5	0.55	0.16 to 1.90	0.348
> 5	0.08	0.02 to 0.32	**< 0.001**^**a**^
Profession			
Registered nurses	1	−	−
Doctors	1.32	0.24 to 7.38	0.751
Midwives	3.16	0.81 to 12.34	0.098
Auxiliary nurses	1.59	0.40 to 6.33	0.506
CHNs	1.9	0.40 to 9.04	0.418
Health/ward assistants	1.76	0.46 to 6.77	0.411
AHW/Pharmacists	1.02	0.24 to 4.29	0.975
Type of facility			
Hospital	1	−	−
Health centre/clinic	1.02	0.30 to 3.51	0.973
District			
Akwapim North	1	−	−
Upper Manya Krobo	5.92	2.10 to 16.67	**0.001**^**a**^
Kwahu West	4.36	2.00 to 12.31	**0.001**^**a**^

^a^statistical significance *P* < 0.05; ^b^Wald-test.

^c^reported per five year increase in age.

AHW: allied health worker; CHN: community health nurse; CHPS: Community-based Health Planning and Services.

Table 5 shows the results of the multivariable logistic regressions for each of the dimensions of motivation and job satisfaction controlling for demographic and work-related factors. Odds ratios with significance below 1 indicated an increase in motivation and job satisfaction and simultaneously a lower likelihood for intention to leave. With regard to the dimensions of motivation significantly associated with turnover intention, job satisfaction (OR = 0.30, 95% CI: 0.12 to 0.73) and intrinsic job satisfaction (OR = 0.32, 95% CI: 0.12 to 0.87) had the lowest odds ratios, while burnout (OR = 0.59, 95% CI: 0.39 to 0.91) and general motivation (OR = 0.60, 95% CI: 0.38 to 0.96) had the highest odds ratios. Concerning the dimensions of job satisfaction significantly associated with turnover intention, satisfaction with tasks (OR = 0.34, 95% CI: 0.17 to 0.65) and morale (OR = 0.40, 95%: 0.20 to 0.83) had the lowest odds ratios, while satisfaction with career development (OR = 0.56, 95% CI: 0.36 to 0.86) and workload (OR = 0.58, 95% CI: 0.34 to 0.99) had the highest odds ratios.

**Table 5:** Adjusted odds ratios (AORs) for the effect of job satisfaction and motivation subscales on turnover intention

**Subscales**	**AOR**	**95% CI**	***P***^**c**^
Motivation subscales			
Job satisfaction	0.30	0.12 to 0.73	**0.008**^**a**^
Burnout	0.59	0.39 to 0.91	**0.017**^**a**^
General motivation	0.60	0.38 to 0.96	**0.034**^**a**^
Organizational commitment	0.36	0.19 to 0.66	**0.001**^**a**^
Intrinsic job satisfaction	0.32	0.12 to 0.87	**0.026**^**a**^
Timeliness and attendance	0.95	0.44 to 2.03	0.885
Conscientiousness	0.63	0.18 to 2.28	0.483
Job satisfaction subscales^**b**^			
Remuneration	0.73	0.45 to 1.18	0.199
Career development	0.56	0.36 to 0.86	**0.008**^**a**^
Management	0.51	0.30 to 0.84	**0.008**^**a**^
Work environment	0.88	0.49 to 1.59	0.675
Workload	0.58	0.34 to 0.99	**0.047**^**a**^
In-service training	0.83	0.53 to 1.33	0.453
Tasks	0.34	0.17 to 0.65	**0.001**^**a**^
Supervision	0.59	0.33 to 1.05	0.072
Morale	0.40	0.20 to 0.83	**0.013**^**a**^

^a^statistical significance *P* < 0.05; ^c^Wald-test.

^b^all subscales refer to satisfaction with the dimensions under review;

^c^Wald-test.

## Discussion

Our study in Ghana showed that motivation and job satisfaction were significantly associated with turnover intention and that higher levels of both reduced the risk of health workers having this intention. Health workers in the study districts achieved a good mean score of overall motivation and a moderate mean score of overall job satisfaction. Moderate mean scores were also achieved in most of the motivational outcomes, but health workers rated themselves generally positive in ‘timeliness and attendance’ and ‘conscientiousness’. The good mean scores achieved in these two areas, however, should be taken with caution, as it is possible that they are subject to social desirability bias in a way that respondents were reluctant to denote themselves negative attributes, such as being inefficient or reporting late to work.

With regard to job satisfaction, health workers in the study districts were least satisfied with their remuneration, career development, management and work environment. Dissatisfaction with these determinants was also reported in similar studies conducted in sub-Saharan Africa in recent years [15,19,30,44,45]. Even though health workers in Ghana belong to the best paid in Western Africa [46], salary levels, including benefits, are still regarded as being too low - a perception, which is also shared by health workers in other parts of Ghana [47,48].

Concerning dissatisfaction with career development, it was suggested that this has much to do with the current promotion practices in Ghana which seem to favour health workers from the urban centres over those from rural areas [18,30]. In a qualitative study conducted by Snow *et al*. [49] in Ghana, health workers also mentioned disadvantages in career development as a major disincentive to accept postings to rural areas. Dissatisfaction with the work environment was mainly related to the availability of medical and technical equipment and the condition of workplaces. Agyepong *et al*. [30] stressed the lack of essential tools and equipment as being among the major workplace obstacles for health workers in the public health sector. They suggest, however, that this has less to do with the absolute unavailability of resources, but rather with a lack of awareness and with capacity regarding supplies management and maintenance.

Health workers achieved a good mean score with regard to intrinsic job satisfaction, such as believing that the own work is valuable, or that something worthwhile is accomplished in the job. According to Franco *et al*. [24], these factors belong to the higher-order motivating factors, meaning factors that stimulate worker motivation even in the absence of extrinsic rewards. Intrinsic job satisfaction primarily impacts on motivation, when extrinsic needs, such as good working conditions or a satisfactory salary, have been met, but also when the work is intrinsically rewarding, for instance, when it provides high social recognition or recognition of achievement by supervisors or colleagues. In other words, being intrinsically satisfied with one’s job may also lead to improved motivation even when health workers are not satisfied with extrinsic work-related factors. This offers an explanation as to why overall motivation was rather good even though health workers were dissatisfied with extrinsic factors, especially with regard to their remuneration, career development and work environment.

Although satisfaction with remuneration was low among the surveyed health workers, it had not been identified as a determinant of turnover intention in this study. In Ghana’s public health sector there is no difference in base salaries between urban or rural areas. In addition, implementation of monetary and non-monetary incentives for health workers working in rural and remote areas have been seriously neglected by the government, even though experiments with such schemes, such as deprived area allowances and car incentives, have been conducted [50,51]. Because remuneration often remains the same also when health workers are transferred to other health facilities, remuneration alone does not seem to provide the necessary condition for health workers in Ghana having turnover intentions. This assumption is supported by Antwi and Philips [52] who found that salary in Ghana is only a determinant of attrition for those health workers who seek employment abroad.

The results in our univariate analysis showed that being a health/ward assistant, aged older than 39 years of age, and working more than 5 years in the current health facility decreased significantly the odds for turnover intention. Health/ward assistants are typically recruited locally and it can be assumed that older health workers are often able in the course of their professional career to get posted close to their spouses, children and/or other family members. Thus, these categories of health workers are often rooted around the workplace through social and family relations and, in consequence, may have a strong rationale to keep their workplace. This was supported by the finding that the odds of having turnover intentions were much higher for health workers who lived separated from their families.

In our multivariate analysis, however, age and the health/ward assistant category were no longer significantly associated with turnover intention. We also did not find statistical evidence for differences in turnover intention among the other surveyed categories of health workers. Although there are a number of studies on turnover intention and turnover that involved different categories of health workers in sub-Saharan Africa [45,53-55], we have identified only two studies that assessed the association between turnover intention and different cadres in this region through regression analysis, but the authors also did not find evidence for differences in this intention in their multivariate analyses [21,56]. However, we did find evidence for the association between turnover intention and district as the place of work. The risk of health workers having turnover intentions was greatest in Upper Manya Krobo, which is an underdeveloped rural and remote district. This finding adds to the evidence that health workers are reluctant to work in such areas, especially in countries with insufficient rural incentive schemes, which has led to the current imbalance of health workers in favour of urban centres in many parts of the world [57,58].

With regard to HRM, the ‘decision space’ of district health managers is limited in Ghana, as key factors for retention, such as salary, access to further education and promotions are under the authority of the national and regional administration. However, district managers do have a role to play: they recommend district staffs for study courses and promotions to their superiors, which both usually lead to higher salaries after completion. Beside recommendations, it is in the authority of district managers to decide on intra-district postings, to conduct supervision and performance appraisals, and to conduct in-service training [59-62]. Evidence compiled by the WHO [1] suggests that these ‘decision spaces’, if performed effectively, positively influence workplace stability through improved motivation and job satisfaction.

Our findings, therefore, suggest that many of the identified motivational outcomes and job satisfaction determinants significantly associated with turnover intention can be influenced by DHMTs. Such factors include job satisfaction, organizational commitment, and satisfaction with management, career development, workload, tasks, and morale. For instance, regular in-service training that focuses on the expressed needs of rural health workers does not only improve staff competence, but it enables health workers to achieve personal goals of career advancement, increases morale and organizational commitment through an improved sense of belonging, and may contribute to reduce workload [1,11,14,29]. Moran *et al*. [63] also found in a recent review on health worker support strategies that access to in-service training contributes to confidence in practicing in rural and remote areas, strongly alleviates professional isolation, and improves retention. Another example is supportive supervision, which is regarded as a key element of HRM for improving motivation, job satisfaction, performance and retention in rural areas [3,11,12].

The position of the District Director of Health Services is ideally held by purpose-trained administrative personnel with qualifications in management and public health. In Ghana, however, many district directors lack these qualifications, as they are often clinicians who did not receive formal management and leadership training [59]. Furthermore, many DHMTs in rural areas are severely understaffed and frequently lack HR officers and other essential managerial staffs, whose tasks are carried out by present team members in addition to their own duties. As a consequence, rural DHMTs in Ghana often lack the skills as well as the capacity to adequately managing their districts, which is reflected in the motivation and job satisfaction outcomes described in our study.

### Limitations

This study was designed as a cross-sectional study of health worker motivation, job satisfaction and turnover intention, and thus provides only a snapshot of health workers’ perspectives at one point in time. The causal relationships between motivation, job satisfaction, and turnover intention, therefore, cannot be further investigated with a cross-sectional design. The data might also be affected by moderacy bias as well as social desirability bias, because extreme answers to questions were regularly avoided and some results indicated that respondents might have had a tendency to answer questions in a way that would be viewed favourably by the interviewer. In addition, our decision to not follow up health workers who had been absent from their workplaces during data collection might have introduced selection bias, because those could have been the less motivated and less satisfied staff.

## Conclusions

This study has demonstrated the link between motivation, job satisfaction and turnover intention in Ghana. Although the HR ‘decision spaces’ at district level in Ghana are limited, our findings indicate that adequate HRM may have the potential to influence motivation and job satisfaction, which in turn will make health workers more likely to remain at their current position. However, district managers in Ghana have typically undergone clinical training and had only limited exposure to general management and HRM. In consequence, there is a need for specific trainings and capacity building measures in this area so that district managers can adequately cope with the requirements and daily tasks of the position they hold.

So to increase motivation and job satisfaction of health workers, district managers should give emphasis on an enabling environment for example through listening to and acting on staff problems and priorities or fostering team building. They may also engage in assisting the career planning and paths of their subordinates. In-service training that is focused on the expressed needs of health workers should be conducted. A supportive supervision system should also be developed that includes experienced and dedicated health workers as supervisors. As such measures are promising in terms of improving motivation and job satisfaction, it is, therefore, worth strengthening HRM skills at district level and supporting district health managers to implement retention strategies.

## Abbreviations

AHW: allied health worker; AOR: adjusted odds ratio; CHAG: Christian Health Association of Ghana; CHN: community health nurse; CHPS: Community-based Health Planning and Services; DHMT: District Health Management Team; GHS: Ghana Health Service; HR: human resource; HRM: human resource management; HS: health system; JDI: Job Descriptive Index; LMICs: low- and middle-income countries; LSTM: Liverpool School of Tropical Medicine; MJS: Measure of Job Satisfaction; OR: odds ratio; WHO: World Health Organization.

## Competing interests

The authors declare that they have no competing interests.

## Authors’ contributions

MB designed and implemented the study, performed the statistical analysis, and drafted the manuscript. MA and PA participated in the design of the study, contributed in its implementation, and commented on draft versions of the manuscript. KW participated in the design and coordination of the study and helped to draft the manuscript. All authors read and approved the final manuscript.

## Supplementary Material

Additional file 1 Motivation: constructs, items and item mean scores. Click here for file

Additional file 2 Job satisfaction: constructs, items and item mean scores. Click here for file
