# Supplementary material for: The effects of health worker motivation and job satisfaction on turnover intention in Ghana: a cross-sectional study
Source: Hum Resour Health. 2014 Aug 9;12:43. doi: 10.1186/1478-4491-12-43 (PMC4130118; doi:10.1186/1478-4491-12-43)
Supplement: Additional file 1 — Motivation: constructs, items and item mean scores. [file 1478-4491-12-43-S1.docx]

| **Constructs** | **Items** | **Mean score^*^ (1-5)** |
| --- | --- | --- |
| 1 General motivation | These days, I feel motivated to work as hard as I can | 2.68 |
|  | I only do this job so that I get paid at the end of the month | 3.98^**^ |
| 2 Burnout | I feel emotionally drained at the end of every day | 3.21^**^ |
|  | Sometimes when I get up in the morning, I dread having to face another day at work | 3.34^**^ |
| 3 Job satisfaction | Overall job satisfaction score | 3.17 |
| 4 Intrinsic job satisfaction | I do not think that my work in my health facility is valuable these days | 4.04^**^ |
|  | I believe that I accomplish something worthwhile in this job | 4.01 |
| 5 Organisational commitment | I am proud to be working for this health facility | 3.71 |
|  | I find that my values and this health facility's values are very similar | 3.39 |
|  | I am glad that I work for this health facility rather than other facilities in the country | 2.85 |
|  | This health facility really inspires me to do my very best on the job | 3.49 |
| 6 Conscientiousness | I always complete my tasks efficiently and correctly | 4.18 |
|  | I am a hard worker | 4.58 |
|  | I do things that need doing without being asked or told | 4.34 |
| 7 Timeliness and attendance | I am punctual about coming to work | 4.23 |
|  | I am often absent from work | 4.42^**^ |
|  | It is not a problem when I sometimes come late to work | 3.78^**^ |
| *^*^a higher score indicates higher levels of motivation ^**^negatively worded questions: a higher score indicates disagreement to these statements* | | |

**Motivation: constructs, items and item mean scores**
