# Supplementary material for: The effects of health worker motivation and job satisfaction on turnover intention in Ghana: a cross-sectional study
Source: Hum Resour Health. 2014 Aug 9;12:43. doi: 10.1186/1478-4491-12-43 (PMC4130118; doi:10.1186/1478-4491-12-43)
Supplement: Additional file 2 — Job satisfaction: constructs, items and item mean scores. [file 1478-4491-12-43-S2.docx]

**Job satisfaction: constructs, items and item mean scores**

| **Constructs^*^** | **Items^**^** | **Mean score**^†^ **(1-5)** |
| --- | --- | --- |
| 1 Remuneration | salary | 2.30 |
|  | benefits | 2.00 |
| 2 Work environment | medical and technical equipment | 2.37 |
|  | physical condition of work place | 2.75 |
|  | availability of drugs | 3.04 |
|  | availability of consumables | 3.31 |
|  | protection against occupational risks | 2.89 |
|  | availability of stationeries | 3.21 |
| 3 Workload | work schedule | 3.22 |
|  | workload | 2.67 |
|  | distribution of the work between members of your team | 3.36 |
|  | distribution of the work between care and your other tasks | 3.22 |
| 4 Tasks | variety of tasks | 3.49 |
|  | match between skills and tasks | 3.68 |
|  | level of professional responsibilities | 3.93 |
| 5 Supervision | quality of interaction with supervisor | 3.80 |
|  | support from supervisor | 3.75 |
|  | recognition of quality of work by supervisor | 3.94 |
| 6 In-service training | in-service training | 3.71 |
|  | the way members of service are selected to participate in  training activities | 3.41 |
| 7 Management | application of rewards in your service in general | 2.02 |
|  | opportunities to participate in decision making to solve problems of work organisation | 3.04 |
|  | information given about the life of your service | 2.97 |
|  | information given about the life of your health facility | 3.04 |
| 8 Career development | ability to advance career | 2.84 |
|  | criteria in place for career advancement | 2.41 |
| 9 Morale | quality of care for patients in your service | 3.61 |
|  | quality of your own work | 4.07 |
|  | recognition of work by patients | 3.85 |
|  | recognition of quality of work by colleagues | 3.99 |
| *^*^all constructs refer to satisfaction with the dimensions of job satisfaction under review*  *^**^all items refer to satisfaction with these items*  ^†^*a higher score indicates higher levels of job satisfaction* | | |
